# Prevalence of Metabolic Syndrome: Association with Risk Factors and Cardiovascular Complications in an Urban Population

**DOI:** 10.1371/journal.pone.0105056

**Published:** 2014-09-02

**Authors:** Gisela Cipullo Moreira, José Paulo Cipullo, Luiz Alberto Souza Ciorlia, Cláudia Bernardi Cesarino, José Fernando Vilela-Martin

**Affiliations:** Internal Medicine Department, Hypertension Clinic, State Medical School in São José do Rio Preto (FAMERP), São Paulo, Brazil; University of Tolima, Colombia

## Abstract

**Introduction:**

Metabolic syndrome (MS) is a set of cardiovascular risk factors and type 2 diabetes, responsible for a 2.5-fold increased cardiovascular mortality and a 5-fold higher risk of developing diabetes.

**Objectives:**

1-to evaluate the prevalence of MS in individuals over 18 years associated with age, gender, socioeconomic status, educational levels, body mass index (BMI), HOMA index and physical activity; moreover, to compare it to other studies; 2-to compare the prevalence of elevated blood pressure (BP), high triglycerides and plasma glucose levels, low HDL cholesterol and high waist circumference among individuals with MS also according to gender; 3-to determine the number of risk factors in subjects with MS and prevalence of complications in individuals with and without MS aged over 40 years.

**Methods:**

A cross-sectional study of 1369 Individuals, 667 males (48.7%) and 702 females (51.3%) was considered to evaluate the prevalence of MS and associated factors in the population.

**Results:**

The study showed that 22.7% (95% CI: 19.4% to 26.0%) of the population has MS, which increases with age, higher BMI and sedentary lifestyle. There was no significant difference between genders until age ≥70 years and social classes. Higher prevalence of MS was observed in lower educational levels and higher prevalence of HOMA positive among individuals with MS. The most prevalent risk factors were elevated blood pressure (85%), low HDL cholesterol (83.1%) and increased waist circumference (82.5%). The prevalence of elevated BP, low HDL cholesterol and plasma glucose levels did not show significant difference between genders. Individuals with MS had higher risk of cardiovascular complications over 40 years.

**Conclusion:**

The prevalence of MS found is similar to that in developed countries, being influenced by age, body mass index, educational levels, physical activity, and leading to a higher prevalence of cardiovascular complications after the 4th decade of life.

## Introduction

Metabolic syndrome (MS) is a set of metabolic disorders that represent risk factors for cardiovascular disease (CVD), atherosclerosis and diabetes mellitus type 2 (DM-2).

High blood pressure (BP) is a frequent component of MS, often associated with insulin resistance and central obesity [1], [2] and MS is an independent predictor of cardiovascular risk in hypertensive patients [3]. Over 85% of individuals with MS have high BP or systemic arterial hypertension (SAH). Hypertensive patients without clinical evidence of CVD show event rates directly related to the number of risk factors for MS. Therefore the presence of MS increases the risk of cardiovascular events by 2-fold and the risk of developing DM-2 [4], [5] by 5-fold.

Insulin resistance is a determining factor in the association among obesity, diabetes, metabolic syndrome and atherosclerotic cardiovascular disease [6], [7]. Obesity, especially visceral fat, is an important link among the components of the syndrome [8], because visceral fat is highly active considering the metabolic aspect. It is also more susceptible to lipolysis compared with subcutaneous fat and is associated with systemic inflammatory response [9]. The HOMA index (Homeostatic Model Assessment) is an alternative for the assessment of insulin resistance, mainly because it is a fast, easily applicable in epidemiological studies and low-cost method. The HOMA index has proved be a robust clinical and epidemiological tool in descriptions of the pathophysiology of diabetes. HOMA analysis allows assessment of inherent β-cell function and insulin sensitivity and can characterize the pathophysiology in those with abnormal glucose tolerance [10].

It has been demonstrated that the prevalence of MS is increasing worldwide, and for the adult population is estimated to be about 20 to 25% [11], largely due to several factors, such as ageing of the population, increased life expectancy and obesity, sedentarism and inadequate nutrition [12]. Studies performed in Latin American populations showed a high prevalence of MS which ranged from 12.3% to 42.7%, depending on the criteria for clinical diagnosis and the characteristics of the study population [13].

Despite of the importance of MS in the context of metabolic and cardiovascular disease, in Brazil, few studies have described the prevalence of MS and its determinants. Thus, we found scarce information on the importance of this problem in our country. Therefore, this study aimed to evaluate the prevalence of metabolic syndrome in an urban adult population, relating it to demographic, anthropometric and biochemical parameters and comparing it to national and international studies. Moreover, it studied association of MS with risk factors and cardiovascular complications.

## Materials and Methods

This project was approved by the Research Ethics Committee of the State Medical School in São José do Rio Preto, São Paulo State, Brazil [14]. All participants were informed about the purpose of the work and provided informed consent before they participated in the study. This was a cross-sectional, population-based study with simple random sampling and stratified by age group in an urban population, with the objective to estimate the prevalence of metabolic syndrome and associated factors in the adult population (≥18 years).

This study was performed in the period of 2004 and 2005. Participants answered a questionnaire with personal data, socioeconomic levels, number of years of schooling, lifestyle, cardiovascular complications (angina, stroke, myocardial infarction, heart failure, coronary bypass). The sample was stratified by age groups of 18–39, 40–49, 50–59, 60–69 and over 70 years old. A total of 1,369 individuals who were submitted to the interviews and laboratory tests were selected from the original sample of 1,717 individuals. 667 of them were men (48.7%) with average age 55.1±14.9 years and 702 of them were women (51.3%) with average age 55.0±14.4 years [14]. The parameters used to calculate the strata sample sizes were number of inhabitants, expected prevalence of hypertension and MS for each age group, maximum allowed 95% confidence interval with semi amplitude of 3% [14].

The urban area was divided into census sectors and the number of individuals studied from each sector was proportional to the population of that area. In each sector, the district, street, and house an adult (resident for more than 6 months) was chosen at random observing the inclusion and exclusion criteria of the study. After the visit to the first house, houses on alternating sides of the street, skipping two residences, were visited. In cases of refusal to participate in the study, an adult resident in the neighboring house was randomly chosen. Exclusion criteria included pregnancy, severe degenerative diseases, incapacitating mental disorders, severe psychiatric diseases, mental deficiency and bedridden patients [14].

Physicians subsequently evaluated the interviews, measured pulse rate and BP, and assessed anthropometric data (weight, stature).

Blood pressure was measured in compliance with standard techniques of the Joint National Committee and Brazilian Guidelines for Hypertension [15], [16].

After 12 hours fasting, blood samples were taken for biochemical tests of blood glucose, total cholesterol (TC), high density lipoprotein cholesterol (HDL-c), triglycerides (TG) and plasma insulin [14].

The socioeconomic level was initially classified as A, B, C, D and E according to the family's possessions and income. Later, 3 levels were adopted for statistical analysis [17]: AB (A+B), C and DE (D+E). The educational level was classified into two groups: NMI: no education to incomplete secondary education (0 to <11 years of schooling); NMCS: complete secondary or higher education (≥11 years of schooling).

Physical activity was assessed by means of a modified International Physical Activity Questionnaire (IPAQ); classifying individuals as active and very active (if they exercised more than 150 minutes per week, including walking, running, swimming and cycling) and inactive or minimally active (if they did not exercised at all or exercised up to 150 minutes per week).

Individuals were also classified according to alcohol consumption into the following categories: no alcohol consumption, moderate alcohol consumption (consumption of ethanol ≤210 grams/week) and high alcohol consumption (consumption exceeding 210 grams/week).

Body mass index (BMI) was calculated as weight in kilograms divided by the square of the height in meters (kg/m^2^). A calibrated portable weighing scale was used to measure the weight. The height was measured using a metric tape. The participants were classified according to their BMI as following: normal weight (BMI<25), overweight (BMI≥25–29.9) and obese (BMI≥30 kg/m^2^). The waist circumference was measured with a tape measure using half the distance between the iliac crest and the lower costal margin [18]. HOMA-IR was calculated by using the following formula:
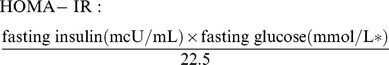
Insulin resistance was defined as a value greater than 2.71 [19]. The HOMA index was evaluated in 840 individuals, which were submitted to dosage of insulin. The insulin evaluation was not performed in another subjects for technical reasons.

The classification adopted for the MS study was defined by the National Cholesterol Education Program-Adult Treatment Panel III (NCEP-ATP III) [5] and updated in 2009, with the following diagnostic criteria: waist circumference (ethnic-specific criteria), TG≥150 mg/dl, HDL-c [<40 mg/dL (male) and <50 mg/dL (female)], BP (SBP≥130 or DBP≥85 mmHg), fasting glucose (≥100 mg/dL or DM-2), or in use of specific treatment for these conditions [20].

The cutoff points for waist circumference utilized were: men ≥102 cm and women ≥88 cm because Caucasians constitute the majority of the studied population (71.58%) [21], and there is no clear classification of the Brazilian population in racial terms.

Therefore, the prevalence of MS and its distribution was evaluated according to age, gender, socioeconomic status, education, physical activity, body mass index (BMI), high waist circumference and MS diagnostic criteria. The relationship among MS and dyslipidemia (changes in TG and HDL-c levels), alcohol consumption, blood glucose, blood pressure, HOMA index and cardiovascular complications were also evaluated.

### Statistical method

The results of this study were corrected for the population. To assess the association between age and MS in both genders, with/without complications, and overall, the likelihood ratio test for independent samples was applied. The comparison between two age levels was performed using the Bonferroni correction for the significance level (α = 0.05/10 = 0.005 comparisons/comparison), i.e., the α Bonferroni = 0.005 [22].

At the population level, sampling was done by age. We also used the method of weighted least squares to analyze the association between MS with all variables (hypertension, cardiovascular complications, socioeconomic status, education level, gender, high waist circumference, BMI, physical activity, number of factors of metabolic syndrome in individuals with the syndrome, HOMA, triglycerides, glucose, HDL-c) were analyzed.

The analysis of HDL-c in relation to alcohol consumption was performed by means of bootstrap simulation method of convex combinations with the same weights used for the analysis of frequencies, where 1000 bootstrap samples were generated for each comparison. The level of significance was α = 0.05 [23].

## Results

The results for the different parameters evaluated are presented by age and corrected for the population, as shown in Table 1.

**Table 1:** Demographic, anthropometric and lifestyle characteristics of studied population.

Characteristics		N	%		N	% corrected for the population
Gender	Male	667	49.7		211	23.3
	Female	702	50.3		256	22.7
Socioeconomic status	AB	288	17.8	92		22.2
	C	584	45.4	195		21.5
	DE	497	36.8	180		25.2
Education (schooling years)	<11 years	989	60.4	363		27.7
	≥11 years	380	39.6	104		15.9
BMI	Normal-weight	538	44.2	75		7.6
	Overweight	511	32.7	196		23.7
	Obese	320	23.1	196		51.6
Physical Activity	Inactive	924	66.9	353		26.1
	Active	445	33.1	114		16.7

All the data are corrected for the total population of city. MS = Metabolic Syndrome; BMI = Body Mass Index.

**Figure 1:**
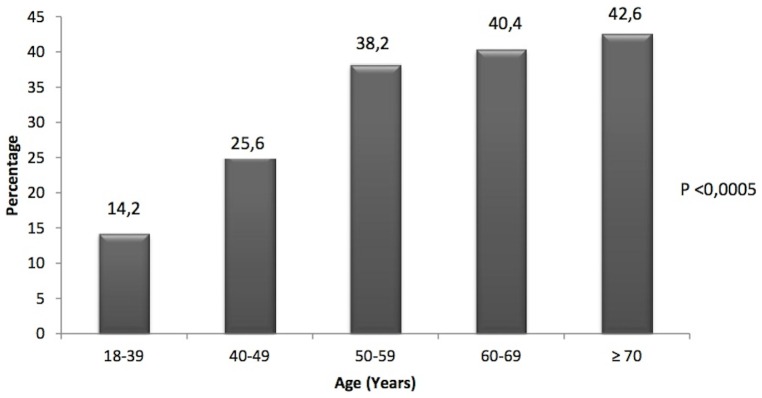
Prevalence of Metabolic Syndrome according to age. It was observed that the prevalence of MS increased with aging years without a linear increase, but with a significant difference among age groups (p<0.0005). The percentage of individuals with MS in each age group was: 14.2% for 18–39 years, 25.6% for 40–49 years, 38.2% for 50–59 years, 40.4% for 60–69 years and 42.6% for those aged ≥70 years.

The prevalence of MS was 22.7% (95% CI: 19.4% to 26.0%), increasing with aging years, without a linear increase; but with a significant difference among age groups (p<0.0005). The percentage of individuals with MS in each age group was: 18–39 years (14.2%), 40–49 years (25.6%), 50–59 years (38.2%), 60–69 years (40.4%) and those aged ≥70 years 42.6%. The prevalence was 3 times greater among elderly compared to younger patients. The figure 1 shows the prevalence of MS according to age.

### Gender

The prevalence of MS in women was 22.7% (95% CI: 18.6% to 27.8%) in men and 23.3% (95% CI: 18.8% to 28.9%). The women/men prevalence ratio was 0.98 (95% CI: 0.72 to 1.32; p = 0.44), with no significant differences among different age groups, except for age ≥70 years (65.4% in women and 34.6% in men; p = 0.0005).

### Socioeconomic Level

The prevalence of MS according to socioeconomic status was: class AB 22.2% (95% CI: 16.9% to 29.2%), class C 21.5% (95% CI: 17.1% to 27.1%) and class DE 25.2% (95% CI: 19.6% to 32.4%). The prevalence ratios were: C/AB: 0.97 (95% CI: 0.67 to 1.39; p = 0.87), DE/AB: 1.14 (95% CI: 0.78 to 1.65; p = 0.51), and DE/C: 1.17 (95% CI: 0.83 to 1.65; p = 0.37).

### Education

The prevalence of MS according to the level of education was: 27.7% (95% CI: 23.4% to 32.6%) up to incomplete secondary level and 15.9% (95% CI: 11.8% to 21.5%) for complete secondary level or higher. The prevalence ratio between NMI/NMCS was: 1.73 (95% CI: 1.23 to 2.45; p = 0.002). It was also observed 73% higher prevalence of MS in individuals with lower educational level.

### BMI

The prevalence of MS according to BMI was: 7.6% (95% CI: 5.1% to 11.3%) in normal-weight subjects, 23.7% (95% CI: 19.0% to 29.5%) in overweight individuals and 51.6% (95% CI: 42.9% to 62.1%) in obese subjects. The prevalence ratios were overweight/normal weight: 3.13 (95% CI: 1.96 to 4.98; p<0.0005), obese/normal weight: 6.8 (95% CI: 4.36 to 10.7; p<0.0005) and obese/overweight: 1.63 (95% CI: 2.91 to 5.28; p<0.0005).

### HOMA-Index

**Figure 2:**
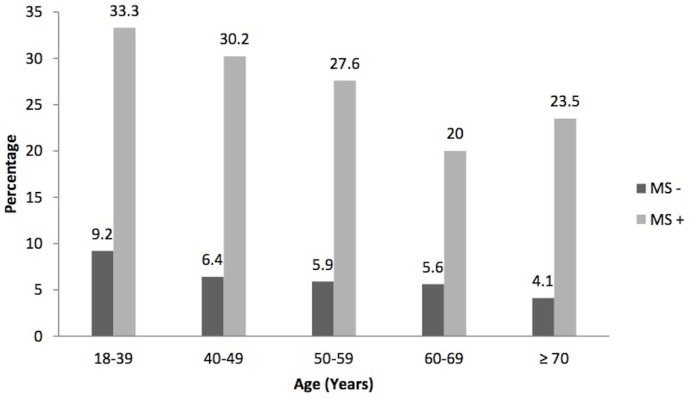
Prevalence of Homa Index+ and Metabolic Syndrome. The figure shows the prevalence of positive HOMA index according to age in individuals with (MS+ = clear bars) and without (MS− = dark bars) metabolic syndrome. The calculation of the HOMA index was performed in 841 individuals, because the insulin value was not determined in all the participants. It was observed that in subjects with MS+, in all the ages, the prevalence of positive HOMA index was always higher than in individuals without MS. MS: Metabolic Syndrome.

The calculation of the HOMA index was performed in 841 individuals, because not all subjects showed insulin value. The prevalence of positive HOMA index was: 26.5% (95% CI: 21.0% to 33.5%) in subjects with MS, and in individuals without MS 6.7% (95% CI: 4.7% to 9.5%). The prevalence ratio between positive and negative MS was: 3.96 (95% CI: 2.60 to 6.05; p<0.0001), almost 4 times higher in subjects with MS compared to those without MS. The figure 2 shows the prevalence of positive Homa index and metabolic syndrome.

### Physical Activity

The prevalence of MS in the active or very active was 16.7% (95% CI: 12.3% to 22.7%) and minimally active or inactive: 26.1% (95% CI: 22.1% to 30.9%), with prevalence ratios inactive/active 1.56 (95% CI: 1.10 to 2.23; p = 0.007).

### Alcohol and MS

**Figure 3:**
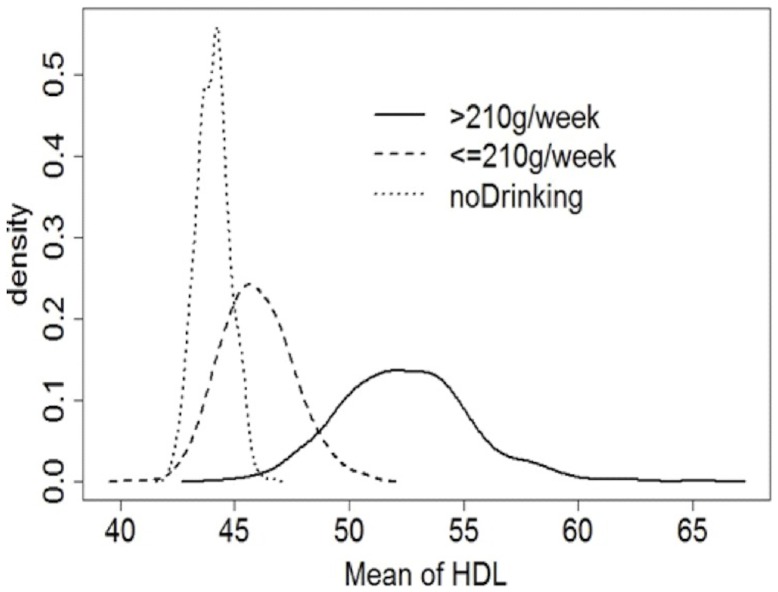
Distribution of HDL-c levels in relation to alcohol consumption. The individuals were divided in according to alcohol consumption into the 3 categories: no alcohol consumption (dotted line), moderate alcohol consumption (≤210 grams/week; dashed line) and high alcohol consumption (>210 grams/week; continuous line). It was observed an association between moderate and/or high alcohol consumption with HDL-c level normal or higher (p<0.0005), while individuals who did not consume alcohol showed a higher percentage of low HDL-C level. The analysis of HDL-c in relation to alcohol consumption was performed by means of bootstrap simulation method.

Regarding the consumption of alcohol, higher consumption was observed in males (p<0.0005). There was an association between moderate and/or high alcohol consumption with HDL-c normal or higher (p<0.0005) (figure 3). Individuals who did not consume alcohol showed a higher percentage of low HDL-c (60.0%). Among subjects who consumed >210 g/week of alcohol, the prevalence of hypertriglyceridemia was 47.8%, and in consumer groups with ≤210 g/week or abstainers, the prevalence was 22.7 and 25.3%, respectively (p<0.0005), with evidence of an association between high alcohol consumption with hypertriglyceridemia.

### MS in hypertensive and normotensive

In this study, the prevalence of MS was 60.4% (95% CI: 54.2% to 67.2%) among hypertensive patients, while only 9.5% (95% CI: 7.0% to 13.0%) of normotensive individuals were affected by the syndrome. The MS prevalence ratio in hypertensive/normotensive patients was 6.32 (95% CI: 4.57 to 8.75; p<0.0005).

### MS components

The evaluation of individuals with MS showed that there was a prevalence of MS components according to gender. Among the population with MS, 85.0% (95% CI: 80.6% to 89.4%) of them showed high blood pressure, 84.8% (95% CI: 79.4% to 90.6%) of whom were women and 85.2% (95% CI: 78.6% to 92.3%) were men, with no difference between genders. The prevalence ratio men/women was 1.00 (95% CI: 0, 91 to 1.11; p = 0.94).

It was observed that 83.1% (95% CI: 79.4% to 86.8%) had levels of lower HDL-c, being 84.2% (95% CI: 79.9% to 88.8%) in women and 81.9% (95% CI: 76.1% to 88.2%) in men. The prevalence ratio men/women was 0.97 (95% CI: 0.89 to 1.07; p = 0.56).

In these individuals, 82.5% (95% CI: 77.9% to 87.0%) had waist measurement above normal, 91.7% (95% CI: 87.2% to 96.4%) in women and 73.1% (95% CI: 65.8% to 81.3%) in men. The prevalence ratio women/men was 1.25 (95% CI: 1.12 to 1.41; p = 0.0001).

The prevalence of changes in TG levels was 69.0% (95% CI: 63.7% to 74.3%) of the individuals, 61.6% (95% CI: 54.4% to 69.8%) in women, and 76 6% (95% CI: 70.1% to 83.9%) in men, with a prevalence ratio men/women of 1.24 (95% CI: 1.07 to 1.45; p = 0.004).

**Figure 4:**
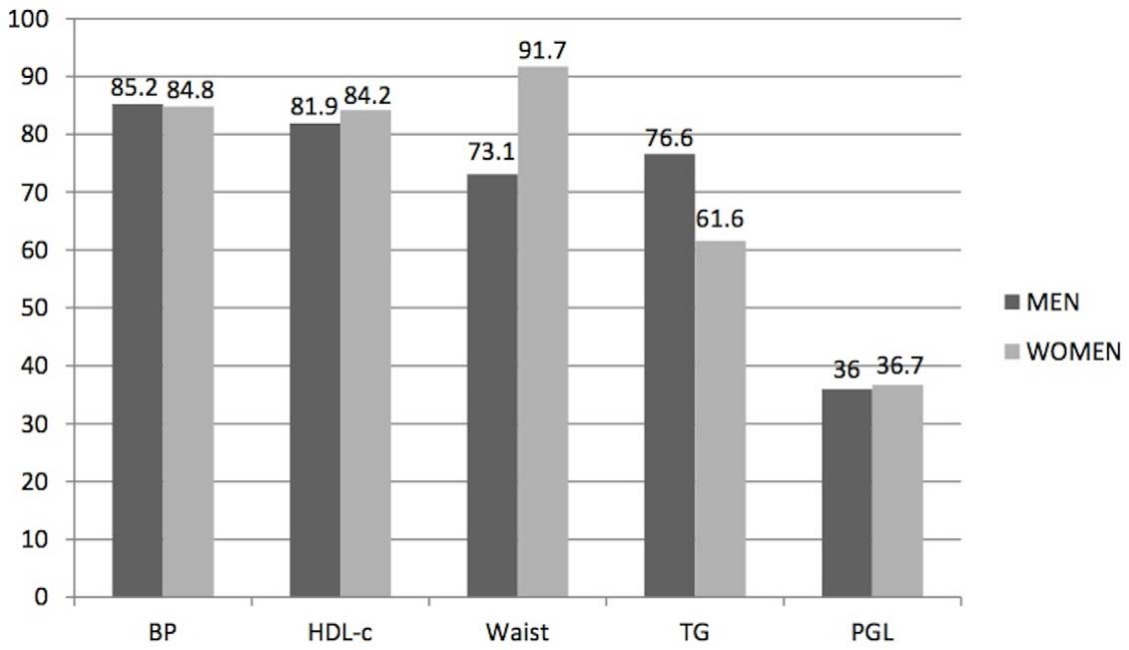
Prevalence of Metabolic Syndrome components according to gender. The evaluation of individuals with metabolic syndrome (MS) showed that there was a different prevalence of MS components according to gender (men = dark bars; women = clear bars). Among the population with MS, 85.0% of them showed high blood pressure (85.2% in men and 84.8% in women). It was observed that 83.1% had low HDL-c levels (81.9% in men and 84.2% in women), while 82.5% had waist measurement above normal (91.7% in women and 73.1% in men). The prevalence of changes in TG levels was 69.0% (76.6% in men and 61.6% in women). Levels of fasting glucose ≥100 mg/dL occurred in 36.4% of the subjects (36.0% in males and 36.7% in females). Blood pressure (BP), HDL-c: HDL-cholesterol, Waist: waist circumference, TG: Triglycerides, PGL: plasma glucose level.

Levels of fasting glucose ≥100 mg/dl occurred in 36.4% (95% CI: 31.3% to 41.5%) of the subjects, 36.7% (95% CI: 30.2% to 44.6%) in females and 36.0% (95% CI: 29.2% to 44.5%) in males. The prevalence ratio male/female was 0.98 (95% CI: 0.73 to 1.31; p = 0.89). The figure 4 shows the prevalence of MS components according to gender.

### Number of diagnostic criteria

Among individuals with MS, 57.7% (95% CI: 50.9% to 66.5%) showed three criteria, 30.2% (95% CI: 23.1% to 37.4%) had four criteria and 11.1% (95% CI: 6.2% to 15.9%) presented five criteria.

### MS prevalence in the population ≥40 years, according to complications

The presence of cardiovascular complications was 11.4% (95% CI: 8.6% to 15.0%) in individuals with the syndrome and 6.1% (95% CI: 4.6% to 8.2%) in those individuals without MS. The prevalence ratios of complications among individuals with and without metabolic syndrome were 1.85 (95% CI: 1.24 to 2.74; p = 0.002).

## Discussion

In this study, it was used the updated NCEP-ATP III criteria as a useful, simple and inexpensive guideline for MS diagnosis, to describe the prevalence of MS in adults according with age, gender, socioeconomic status, educational levels, BMI, HOMA index and physical activity [5]. Another classification usually utilized for definition of MS is based in The International Diabetes Federation (IDF) criteria; however, IDF and ATPIII criteria show a good agreement, reason why was used the updated ATP III classification, without comparisons between them [20]. The population prevalence of MS (22.7%) was very similar to that seen in the American study NHANES (23.7%) [24], maybe because it adopted similar criteria for age distribution and included the urban population only. In Brazil, a systematic review showed a mean prevalence of MS of 29.6% (range: 14.9%–65.3%) [25]. However, the studies were performed in little population samples [25]. Despite the methodological differences (half of these studies used the definition proposed by NCEP-ATP III) and the lack of consensus on criteria for MS diagnosis, this review indicated a high prevalence of MS in the healthy Brazilian adult population. As mentioned in the literature, increased prevalence of MS with age [24], [26] was also observed in the present study.

There were no significant differences between genders in all age groups, except for individuals aged ≥70 years, in which there was a predominance of females (65.4%). Ervin noted that men and women aged 40–59 years were three times more likely to have MS compared with those ranging in age from 20–39 years. In men aged ≥60 years, this probability was 4 times higher, and in women of the same age, the chance was increased by 6 times [27].

In the present study, the prevalence of MS was 3 times higher in elderly subjects (≥60 years) in relation to the group <60 years. It was also 4 times higher among females and 2.2 higher among males. These differences can be explained because in individuals with less than 60 years of age, there were differences regarding susceptibility of MS components among genders, including obesity, insulin resistance, SAH and CVD. The differences in insulin resistance seem to be related to differences in the anatomical distribution of fat: men have a greater amount of visceral fat, linked to insulin resistance, whereas subcutaneous fat is more predominant in women [28].

The prevalence of higher MS in classes DE (25.2%) compared to class AB (22.2%) and C (21.5%), although not statistically significant, shows a trend of higher prevalence in lower socioeconomic levels. Ramsay *et al.* observed inverse relation between social class and MS in adults, which can be explained by several factors, such as physical activity, alcohol consumption and smoking [29]. Cesarino and colleagues demonstrated in São José do Rio Preto (Brazil) that hypertensive adults belonging to working age population are mostly Caucasians and married, have low level of education and belong to the lower social classes [30]. In the present study, the prevalence of MS was 73% higher in individuals with lower educational level. In a Brazilian study, educational level was significantly associated with MS components, hyperglycemia, higher waist circumference and hypertension in women. The level of education is considered a reliable and relevant indicator of social status, especially in relation to women whose housework schedules are not always paid. The biological basis for the association with educational disparities in SM remains unclear. It has been suggested that socioeconomic status influences nutrition and sedentary habits, which are highly related to MS components [31].

It can also be observed, in all age groups, a higher prevalence of MS in overweight and obese individuals. Prevalence of the obesity and the MS is quickly increasing in developing countries, leading to higher morbidity and mortality, according to studies realized in several populations, as shows the table 2. This table presents essential information on MS from similar studies performed in developing countries (prevalence of MS according to different criteria diagnosis, numbers of patients and age range, and comparison among diagnosis criteria of MS used in the studies) [25], [32]–[48]. The burden of overweight and obesity seems to be related to nutrition transition in the last decades. In Brazil, between the periods 1974/75 to 2008/09, it has been observed a reduction of underweight individuals and an increase in the rate of overweight (18.6% to 50.1% in men and 28.6 to 48.0% in women) and obesity (2.8% to 12.8% in men and 7.8 to 16.9% in women). Perhaps this can be explained by a reduction in the consumption of basic foods and increased participation of ultra processed foods in the diet used [49]. So, overweight and obesity is a natural consequence of nutrition and sedentary lifestyle. Increasing burden of obesity, MS, DM-2 and CVD in developing countries has created an urgent need to strategize health policies and mass interventions programs [50]. Thus, the determination of insulin resistance levels is of clinical importance to identifying individuals with high risk of metabolic diseases and atherosclerosis [19]. In this study, the prevalence ratio among individuals with MS/without metabolic syndrome (3.96) shows a lot higher prevalence of positive HOMA index in individuals with MS. The values of insulin resistance are predictors of cardiovascular and atherosclerotic events. Surrogate measures of insulin sensitivity such as HOMA index can be used to measure the levels of glucose and insulin [19].

**Table 2:** Prevalence of the metabolic syndrome in developing countries.

Author/year	Country/region and urban/rural area	Prevalence (%)		Age (yr)	Sample (n)		Criterion for diagnosis
		Male	Female		Male	Female	
Podang J et al, 2013 [32]	Thailand	18.2	10.3	----	1875	669	NCEP-ATPIII
Jaipakdee J et al, 2013 [33]	Thailand	25.1		35–60	628	2176	Joint Statement 2009: (IDF, NHLBI, AHA, WHF, IAS, IASO)
Tamang HK et al, 2013 [34]	Nepal	76.9		26–90	221 (total)		NCEP-ATPIII
Sy RG et al, 2014 [35]	Philippines	19.7	25.6	20–50	3072 (total)		IDF
							NCEP-ATPIII
Gupta et al, 2007 [36]	North India (urban)	22.9	31.6	>20	532	559	NCEP-ATPIII
Deepa et al, 2007 [37]	South India (urban)	23.2		≥20	23505 (total)		WHO
		18.3					NCEP-ATPIII
		25.8					IDF
Chow et al, 2008 [38]	India (rural)	32.5	23.9	≥30	4535 (total)		Modified NCEP-ATPIII
Xi B et al, 2013 [39]	China	21.3		≥18	7488 (total)		NCEP-ATPIII
		18.2					IDF
Liu M et al, 2013 [40]	China	50.4 (2001)		60–95	943	1391 (2001)	Joint Statement 2009: (IDF, NHLBI, AHA, WHF, IAS, IASO)
		58.1 (2010)			848	1254 (2010)	
Xu S et al, 2014 [41]	China	29 - Rural		≥20	3297		Joint Statement 2009: (IDF, NHLBI, AHA, WHF, IAS, IASO)
		25.9 - Urban					
You L et al, 2014 [42]	China (Mongolian Area)	36.7	17.8	20–80	809	617	Joint Statement 2009: (IDF, NHLBI, AHA, WHF, IAS, IASO)
Al-Daghri NM et al, 2013 [43]	Saudi Arabia	39 (total)		19–60	87	98	IDF
		24	55				
Shahini N et al, 2013 [44]	Turkey	35		33.6±6.8 mean±SD	160 (total)		NCEP-ATPIII
Onat A et al, 2013 [45]	Turkey	53		≥40	796 (total)		NCEP-ATPIII
Esmailzadehha N et al, 2013 [46]	Iran	28		20–78	529	578	WHO
		26.2					NCEP-ATPIII (2001)
		30.6					NCEP-ATPIII (2004)
		34.2					IDF
		33					AHA/NHLBI
		39.3					Joint Statement 2009
De Carvalho Vidigal F et al, 2013 [25]	Brazil (10 studies)	29.6		19–64	3334	5171	NCEP-ATPIII
		(14.9–65.3)					
Saad MA et al, 2014 [47]	Brazil	51.9		>60	63	180	WHO modified
		45.2					NCEP-ATPIII
		64.1					IDF
		69.1					Joint Statement 2009
Del Brutto OH et al, 2013 [48]	Ecuador	55.7		≥40	517 (total)		IDF
Moreira GC et al, 2014*	Brazil	23.3	22.7	≥40	667	702	Joint Statement 2009

NCEP-ATPIII = National Cholesterol Education Program-Adult Treatment Panel III; WHO = World Health Organization; IDF = International Diabetes Federation; AHA = American Heart Association; NHLBI = National Heart, Lung, Blood Institute; WHF = World Heart Federation; IAS = International Atherosclerosis Society; and IASO = International Association for the Study of Obesity.

Joint Statement 2009: (IDF, NHLBI, AHA, WHF, IAS, IASO).

SD = standard deviation.

* present study.

In this study, the prevalence of MS was 6 times higher in the hypertensive population, with 14 times greater risk of MS in hypertensive compared with non-hypertensive individuals, indicating a clear association between SAH and other risk factors for CVD. The presence of MS in hypertensive patients significantly increases the risk for CVD, DM-2 and mortality. The NCEP-ATP III classification was used in three European countries and a high prevalence of MS in hypertensive patients was found in 61% of patients in Germany, 22% in Spain and 21% in Italy. The incidence of CVD and mortality was 2 times higher in individuals with MS, and the prevalence of DM-2 was 6 times higher [51].

A study by Franco *et al.* found a high prevalence of MS among hypertensive patients in the city of Cuiabá, significantly associated with BMI>25 Kg/m^2^, with insulin resistance (HOMA index) and especially with a family history of hypertension in the multiple regression analysis [52]. The order of prevalence of MS components in the studied population was: high BP (85.0%), HDL-c (83.1%), high waist circumference (82.5%), TG (69.0%), and blood glucose (36.4%), observing significant differences between genders only in waist circumference values and TG values. Among men, the prevalence order of factors was: high BP (85.2%), HDL-c (81.9%), TG (76.6%), waist circumference (73.1%) and blood glucose (36.0%), and among women: waist circumference (91.7%), high BP (84.8%), HDL-c (84.2%), TG (61.6%) and blood glucose (36.7%). Evaluating the prevalence ratios of MS components between genders, significant differences in waist circumference (more prevalent among women) and TG levels (more prevalent among men) were observed. A study by *Ervin* found that more prevalent components of MS were high waist circumference, SAH and high TG. Among men, high TG, high BP and blood glucose was found; whereas among women, high waist circumference and low HDL-c [27]. These findings were very similar to present study. In a Swedish study, high BP was the most common component in both sexes, followed by high waist circumference among women and changes in TG levels among men [53]. In China, the prevalence of individual components of MS was in order: high BP, TG, plasma glucose, central obesity and HDL-c in men and high BP, central obesity, TG, HDL-c and plasma glucose in women [54]. In a Brazilian systematic review, the most frequent MS components were low HDL-c (59.3%) and high BP (52.5%) [25].

In order to investigate the association of two components of MS (HDL-c and TG) with alcohol use in both genders, 3 levels of alcohol consumption were compared in studied individuals. In individuals with MS, it was found that moderate or high consumption of alcohol was related to higher prevalence of HDL-c normal or increased when compared to abstainers. Prevalence of hypertriglyceridemia was higher among individuals with high alcohol consumption (47.8%) compared with those with moderate consumption (22.7%) and abstainers (25.3%) (p<0.0005); therefore, a higher alcohol consumption in males could explain a higher prevalence of hypertriglyceridemia, as well as the association between moderate/high consumption alcohol with normal or high HDL-c. Despite higher plasma levels of HDL-c with alcohol consumption and the clear prevalence of alcohol consumption among men, there was no significant difference in HDL-c between genders. Epidemiologic studies demonstrate higher levels of HDL-c in alcohol consumers. Alcohol affects lipoprotein metabolism in several stages. Regular consumption may be associated with increased synthesis of lipoproteins, decreased degradation of HDL-c, greater hepatic metabolism of LDL-cholesterol, further increase of triglycerides, with the inhibition of the oxidation of free fatty acids. Alcohol consumption is also responsible for modifying the dynamic metabolism of HDL-c [55]. In South Korean, a study observed significantly higher TG levels among excessive drinkers men. Such high consumption of alcohol was associated with high risk of MS due to high BP, impaired fasting glucose, abdominal obesity and TG [56].

Patients over 50 years showed a higher prevalence of increased mass index (overweight and obese) in women (63.1%) compared to men (36.9%) (p = 0.01), which may explain the highest prevalence of increased waist circumference in women. Ervin also noted greater abdominal obesity in patients over 40 years old [27]. Metabolic syndrome becomes more common with age and body weight increase.

In this study, after analyzing the prevalence ratio between inactive/active patients, it was concluded that inactive individuals were 56% more likely to have MS. A Japanese study showed that moderate-vigorous physical activity was sufficient to reduce the prevalence and risk to develop metabolic syndrome among middle-aged men and women [57]. In Norway, HUNT 2 study demonstrated that among individuals with MS, even low level of physical activity was associated with reduced mortality due to all causes, including cardiovascular diseases [58].

In the studied population, 57.7% of individuals with MS had 3 risk factors, 30.2% of them had four risks and 11.1% of them showed five factors. Kokubo *et al.* showed that the incidence risk for CVD increased according to the number of MS components. The risk was similar among individuals with the same number of components [59].

The prevalence of cardiovascular complications was evaluated in subjects ≥40 years, due to the larger number of events among this age group. Among individuals with MS, 11.4% were affected by complications compared to 6.1% without the syndrome. By analyzing the prevalence ratios of complications among individuals with and without MS, it is concluded that individuals with MS are 50% more likely to be affected by complications. Evidence shows that metabolic and hemodynamic abnormalities of MS are associated with the prevalence of subclinical damage in various organs, as well as with increased risk of fatal cardiovascular events [60]. Individuals with MS have cardiovascular risk 50–60% higher than those without the syndrome [61]. A meta-analysis of 87 studies (951,083 patients) showed that MS was associated with a 2-fold increase in risk for CVD and 1.5-fold increase in all-cause mortality [62]. Patients with MS, but without diabetes, also had high cardiovascular risk.

### Limitations of the study

Some limitations of this study should be mentioned. Firstly, the guidelines of the American Diabetes Association recommend the confirmation of hyperglycemia through a second blood glucose measurement, which was not performed in this study. However, in the epidemiological studies, including NHANES, used almost exclusively a single blood glucose measurement for the diagnosis of DM. Secondly, we did not assess the interference of antilipemic drugs in the lipid values determination. Thirdly, the calculation of HOMA index was not performed in the total samples as not all subjects showed insulin values. Finally, data concerning complications were obtained from the clinical history, without conducting specific tests which evidence that the organic lesion occurred.

On the other hand, this population-based, age-stratified study, with a normotensive control group, is unique as it gathers different demographic, epidemiologic and risk factors involved in the genesis of hypertension and CVD in a single sample with a population assessment calculation, which might be extrapolated to other hypertensive populations.

## Conclusions

The prevalence of MS in this study is similar to that of developed countries: it increases with age, shows no significant differences between sex and among social classes. However, lower levels of education are associated with higher prevalence of MS. Metabolic syndrome is more prevalent in individuals with higher BMI (especially obese) and inactive or minimally active. The positive HOMA Index is more prevalent in individuals with MS. The diagnostic criteria for MS with a higher prevalence are: high blood pressure, low HDL-C and high waist circumference. Diagnostic criteria - high BP, low HDL-C and changes in blood glucose - similarly affect both genders, with no significant differences. There was a higher prevalence of high waist circumference in women, and high TG in men. Individuals aged ≥40 years with MS have a higher prevalence of cardiovascular complications. Therefore, we understand that this is one of the first studies conducted in Brazil addressing different aspects involved in the metabolic syndrome and may serve as a warning for public authorities to control and prevent obesity and hypertension.

## References

[1] Moller DE, Kaufman KD (2005) Metabolic syndrome: a clinical and molecular perspective. Annu Rev Med 56: 45–62. 15660501 10.1146/annurev.med.56.082103.104751

[2] Afonso LC, Edelson GW, Sowers JR (1997) Metabolic abnormalities in hypertension. Curr Opin Nephrol Hypertens 6: 219–223. 9263663 10.1097/00041552-199705000-00004

[3] Pierdomenico SD, Lapenna D, Di Tommaso R, Di Carlo S, Caldarella MP, et al (2007) Prognostic relevance of metabolic syndrome in hypertensive patients at low-to-medium risk. Am J Hypertens 20: 1291–1296. 18047919 10.1016/j.amjhyper.2007.06.011

[4] Franklin SS (2006) Hypertension in the metabolic syndrome. Metab Syndr Relat Disord 4: 287–298. 18370747 10.1089/met.2006.4.287

[5] Grundy SM, Cleeman JI, Daniels SR, Donato KA, Eckel RH, Franklin BA, et al (2005) Diagnosis and management of the metabolic syndrome: An American Heart Association/National Heart, Lung, and Blood Institute Scientific Statement. Circulation 112: 2735–52. 16157765 10.1161/CIRCULATIONAHA.105.169404

[6] Savage DB, Petersen KF, Shulman GI (2005) Mechanisms of insulin resistance in humans and possible links with inflammation. Hypertension 45: 828–833. 15824195 10.1161/01.HYP.0000163475.04421.e4

[7] Balkau B, Valensi P, Eschwége E, Slama G (2007) A review of the metabolic syndrome. Diabetes Metab 33: 405–413. 17981485 10.1016/j.diabet.2007.08.001

[8] Mehta NN, Reilly MP (2004) Mechanisms of the metabolic syndrome. Drug Discovery Today: Disease Mechanisms 1: 187–194.

[9] Bahceci M, Gokalp D, Bahceci S, Tuzcu A, Atmaca S, et al (2007) The correlation between adiposity and adiponectin, tumor necrosis factor α, interleukin-6 and high sensitivity C-reactive protein levels. Is adipocyte size associated with inflammation in adults? J Endocrinol Invest 30: 210–214. 17505154 10.1007/BF03347427

[10] Vasques AC, Rosado LE, Alfenas RCG, Geloneze B (2008) Critical analysis on the use of the homeostasis model assessment (HOMA) indexes in the evaluation of the insulin resistance and the pancreatic beta cells functional capacity. Arq Bras Endocrinol Metabol 52: 32–39. 18345394 10.1590/s0004-27302008000100006

[11] International Diabetes Federation. Information on the IDF consensus worldwide definition of the metabolic syndrome. Available: http://www.idf.org/webdata/docs/IDF_Meta_def_final.pdf. Accessed 2014 Apr 10.

[12] Xavier HT, Monte O (2005) Atherosclerosis prevention in metabolic syndrome patients: from physiopathology to the farmacoeconomics of statins treatment. Rev Bras Med 62: 197–204.

[13] Márquez-Sandoval F, Macedo-Ojeda G, Viramontes-Horner D, Fernández Ballart JD, Salas Salvadó J, et al (2011) The prevalence of metabolic syndrome in Latin America: a systematic review. Public Health Nutr 14: 1702–1713. 21486521 10.1017/S1368980010003320

[14] Cipullo JP, Martin JF, Ciorlia LA, Godoy MR, Cação JC, et al (2010) Hypertension prevalence and risk factors in a Brazilian urban population. Arq Bras Cardiol 94: 488–494. 10.1590/s0066-782x2010005000014 20339819

[15] Chobanian AV, Bakris GI, Black HR, Cushman WC, Green LA, et al (2003) The seventh report of the Joint National Committee on Prevention, Detection, Evaluation, and Treatment of High Blood Pressure. The JNC 7 Report. JAMA 289: 2560–2572. 12748199 10.1001/jama.289.19.2560

[16] VI Diretrizes Brasileiras de Hipertensão (2010) Information on Brazilian Guidelines for Hypertension. Available: http://publicacoes.cardiol.br/consenso/2010/Diretriz_hipertensao_ERRATA.pdf. Accessed 2013 Sep 07.

[17] Krieger N, Williams DR, Moss NE (1997) Measuring social class in U.S. public health research: concepts, methodologies, and guidelines. Annu Rev Publ Health 18: 341–378. 10.1146/annurev.publhealth.18.1.341 9143723

[18] Associação Brasileira para o Estudo da Obesidade e da Síndrome Metabólica (Abeso). Information on Obesity and Metabolic Syndrome in Brazil. Available: http://www.abeso.org.br. Accessed 2013 Sep 09.

[19] Geloneze B, Repetto EM, Geloneze SR, Tambascia MA, Ermetice MN (2006) The threshold value for insulin resistance (HOMA-IR) in an admixtured population. IR in the Brazilian Metabolic Syndrome Study. Diabetes Res Clin Pract 72: 219–220. 16310881 10.1016/j.diabres.2005.10.017

[20] Alberti KGM, Eckel RH, Grundy SM, Zimmet PZ, Cleeman JI, et al (2009) Harmonizing the metabolic syndrome. A Joint Interim Statement of the International Diabetes Federation Task Force on Epidemiology and Prevention; National Heart, Lung, and Blood Institute; American Heart Association; World Heart Federation; International Atherosclerosis Society; and International Association for the Study of Obesity. Circulation 120: 1640–1645. 19805654 10.1161/CIRCULATIONAHA.109.192644

[21] Brazilian Institute for Geography and Statistics (IBGE). Information on population census. Available: http://www.ibge.gov.br/censo/. Accessed 2013 Aug 19.

[22] Bland JM, Altman DG (1995) Multiple significance tests: the Bonferroni method. BMJ 310: 170. 7833759 10.1136/bmj.310.6973.170 PMC2548561

[23] Efron B, Tibshirami RJ (1993) An introduction to the bootstrap. Monographs on statistics and applied probability. New York: Chapman & Hall.

[24] Ford ES, Giles WH, Dietz WH (2002) Prevalence of the metabolic syndrome among US adults: findings from the Third National Health and Nutrition Examination Survey. JAMA 287: 356–359. 11790215 10.1001/jama.287.3.356

[25] De Carvalho Vidigal F, Bressan J, Babio N, Salas-Salvadó J (2013) Prevalence of metabolic syndrome in Brazilian adults: a systematic review. BMC Public Health 13: 1198. 24350922 10.1186/1471-2458-13-1198 PMC3878341

[26] Grundy SM (2008) Metabolic syndrome pandemic. Arterioscler Thromb Vasc Biol 28: 629–636. 18174459 10.1161/ATVBAHA.107.151092

[27] Ervin RB (2009) Prevalence of metabolic syndrome among adults 20 years of age and over, by sex, age, race and ethnicity, and body mass index: United States, 2003–2006. National Health Statistics Reports 13: 1–7. 19634296

[28] Luses AL, Attie AD, Reue K (2008) Metabolic syndrome: from epidemiology to systems biology. Nat Rev Genet 9: 819–830. 18852695 10.1038/nrg2468 PMC2829312

[29] Ramsay SE, Whinaup PH, Morris R, Lennon L, Wannamethee SG (2008) Is socioeconomic position related to the prevalence of metabolic syndrome? Influence of social class across the life course in a population-based study of older men. Diabetes Care 31: 2380–2382. 18809625 10.2337/dc08-1158 PMC2584199

[30] Cesarino CB, Cipullo JP, Martin JF, Ciorlia LA, Godoy MR, et al (2008) Prevalence and Sociodemographic Factors in a Hypertensive Population in São José do Rio Preto, São Paulo, Brazil. Arq Bras Cardiol 91: 29–33. 18660942 10.1590/s0066-782x2008001300005

[31] Gronner MF, Bosi PL, Carvalho AM, Casale G, Contrera D, et al (2011) Prevalence of metabolic syndrome and its association with educational inequalities among Brazilian adults: a population-based study. Braz J Med Biol Res 44: 713–719. 21755260 10.1590/s0100-879x2011007500087

[32] Podang J, Sritara P, Narksawat K (2013) Prevalence and factors associated with metabolic syndrome among a group of Thai working population: a cross sectional study. J Med Assoc Thai 96: 33–41. 24851571

[33] Jaipakdee J, Jiamjarasrangsri W, Lohsoonthorn V, Lertmaharit S (2013) Prevalence of metabolic syndrome and its association with sérum uric acid levels in Bangkok Thailand. Southeast Asian J Trop Med Public Health 44: 512–522. 24050084

[34] Tamang HK, Timilsina U, Thapa S, Singh KP, Shrestha S, et al (2013) Prevalence of metabolic syndrome among Nepalese type 2 diabetic patients. Nepal Med Coll J 15: 50–55. 24592795

[35] Sy RG, Llanes EJ, Reganit PF, Castillo-Carandang N, Punzalan FE, et al (2014) Socio-demographic factors and the prevalence of metabolic syndrome among Filipinos from the LIFECARE cohort. J Atheroscler Thromb 21 Suppl 1: S9–17. 24452117 10.5551/jat.21_sup.1-s9

[36] Gupta R, Sarna M, Thanvi J, Sharma V, Gupta VP (2007) Fasting glucose and cardiovascular risk factors in an urban population. J Assoc Physicians India 55: 705–709. 18173023

[37] Deepa M, Farooq S, Datta M, Deepa R, Mohan V (2007) Prevalence of metabolic syndrome using WHO, ATPIII and IDF definitions in Asian Indians: the Chennai Urban Rural Epidemiology Study (CURES-34). Diabetes Metab Res Rev 23: 127–134. 16752431 10.1002/dmrr.658

[38] Chow CK, Naidu S, Raju K, Raju R, Joshi R, et al (2008) Significant lipid, adiposity and metabolic abnormalities amongst 4535 Indians from a developing region of rural Andhra Pradesh. Atherosclerosis 196: 943–952. 17466992 10.1016/j.atherosclerosis.2007.02.027

[39] Xi B, He D, Hu Y, Zhou D (2013) Prevalence of metabolic syndrome and its influencing factors among the Chinese adults: the China Health and Nutrition Survey in 2009. Prev Med 57: 867–871. 24103567 10.1016/j.ypmed.2013.09.023 PMC4044099

[40] Liu M, Wang J, Jiang B, Sun D, Wu L, et al (2013) Increasing prevalence of metabolic syndrome in a chinese elderly population: 2001–2010. PLoS One 8: e66233. 23824753 10.1371/journal.pone.0066233 PMC3688874

[41] Xu S, Ming J, Yang C, Gao B, Whan Y, et al (2014) Urban, semi-urban and rural difference in the prevalence of metabolic syndrome in Shaanxi province, northwestern China: a population-based survey. BMC Public Health 14: 104. 24484601 10.1186/1471-2458-14-104 PMC3910226

[42] You L, Liu A, Wuyun G, Wu H, Wang P (2014) Prevalence of hyperuricemia and the relationship between serum uric acid and metabolic syndrome in the Asian Mongolian area. J Atheroscler Thromb 21: 355–365. 24401703 10.5551/jat.20529

[43] Al-Daghri NM, Khan N, Alkharfy KM, Al-Attas OS, Alokail MS, et al (2013) Selected dietary nutrients and the prevalence of metabolic syndrome in adult males and females in Saudi Arabia: a pilot study. Nutrients 5: 4587–4604. 24284611 10.3390/nu5114587 PMC3847750

[44] Shahini N, Shahini I, Marjani A (2013) Prevalence of metabolic syndrome in turkmen ethnic groups in gorgan. J Clin Diagn Res 7: 1849–1851. 24179879 10.7860/JCDR/2013/6035.3331 PMC3809618

[45] Onat A, Yüksel M, Köroğlu B, Gümrükçüoğlu HA, Aydın M, et al (2013) Turkish Adult Risk Factor Study survey 2012: overall and coronary mortality and trends in the prevalence of metabolic syndrome. Turk Kardiyol Dern Ars 41: 373–378. 23917000 10.5543/tkda.2013.15853

[46] Esmailzadehha N, Ziaee A, Kazemifar AM, Ghorbani A, Oveisi S (2013) Prevalence of metabolic syndrome in Qazvin Metabolic Diseases Study (QMDS), Iran: a comparative analysis of six definitions. Endocr Regul 47: 111–120. 23889480 10.4149/endo_2013_03_111

[47] Saad MA, Cardoso GP, Martins W de A, Velarde LG, da Cruz Filho RA (2014) Prevalence of metabolic syndrome in elderly and agreement among four diagnostic criteria. Arq Bras Cardiol 102: 263–269. 24676226 10.5935/abc.20140013 PMC3987322

[48] Del Brutto OH, Zambrano M, Peñaherrera E, Montalván M, Pow-Chon-Long F, et al (2013) Prevalence of the metabolic syndrome and its correlation with the cardiovascular health status in stroke- and ischemic heart disease-free Ecuadorian natives/mestizos aged ≥40 years living in Atahualpa: A population-based study. Diabetes Metab Syndr 7: 218–222. 24290088 10.1016/j.dsx.2013.10.006

[49] Jaime PC, Ministério da Saúde (2013) A Nova Política Nacional de Alimentação e Nutrição e os desafios na Promoção da Alimentação Adequada e Saudável no SUS. Information on Brazilian food policy. Available: http://www.cve.saude.sp.gov.br. Accessed 2014 Jun 26.

[50] Misra A, Khurana L (2008) Obesity and the metabolic syndrome in developing countries. J Clin Endocrinol Metab 93 11 Suppl 1: S9–S30. 18987276 10.1210/jc.2008-1595

[51] Scholze J, Alegria E, Ferri C, Langham S, Stevens W, et al (2010) Epidemiological and economic burden of metabolic syndrome and its consequences in patients with hypertension in Germany, Spain and Italy; a prevalence-based model. BMC Public Health 10: 529–540. 20813031 10.1186/1471-2458-10-529 PMC2940918

[52] Franco GP, Scala LC, Alves CJ, França GV, Cassanelli T, et al (2009) Metabolic syndrome in patients with high blood pressure in Cuiabá-Mato Grosso State: prevalence and associated factors. Arq Bras Cardiol 92: 472–478. 10.1590/s0066-782x2009000600010 19629311

[53] Novak M, Bjorck L, Welin L, Welin C, Manhem K, et al (2013) Gender differences in the prevalence of metabolic syndrome in 50-year-old Swedish men and women with hypertension born in 1953. J Hum Hypertens 27: 56–61. 22129609 10.1038/jhh.2011.106

[54] Zhao Y, Yan H, Yang R, Li Q, Dang S, et al (2014) Prevalence and determinants of metabolic syndrome among adults in a rural area of Northwest China. PLoS One 9: e91578. 24614618 10.1371/journal.pone.0091578 PMC3948893

[55] Park H, Kim K (2012) Association of Alcohol Consumption with Lipid Profile in Hypertensive Men. Alcohol and Alcohol 47: 282–287. 10.1093/alcalc/ags019 22371847

[56] Kim J, Chu SK, Kim K, Moon JR (2011) Alcohol use behaviors and risk of metabolic syndrome in South Korean middle-aged men. BMC Public Health 11: 489–496. 21693063 10.1186/1471-2458-11-489 PMC3151233

[57] Kim J, Tanabe K, Yokoyama N, Zempo H, Kuno S (2011) Association between physical activity and metabolic syndrome in middle-aged Japanese: a cross-sectional study. BMC Public Health 11: 624–631. 21819591 10.1186/1471-2458-11-624 PMC3199599

[58] Stensvold D, Nauman J, Nilsen TI, Wisløff U, Slørdahl SA, et al (2011) Even low level of physical activity is associated with reduced mortality among people with metabolic syndrome, a population based study (the HUNT 2 study, Norway). BMC Med 9: 109–116. 21958416 10.1186/1741-7015-9-109 PMC3193808

[59] Kokubo Y, Okamura T, Yoshimosa Y, Miyamoto Y, Kawanishi K, et al (2008) Impact of metabolic syndrome components on the incidence of cardiovascular disease in a general urban Japanese population: the Suita study. Hypertens Res 31: 2027–2035. 19098374 10.1291/hypres.31.2027

[60] Cuspidi C, Sala C, Zanchetti A (2008) Metabolic syndrome and target organ damage: role of blood pressure. Expert Rev Cardiovasc Ther 6: 731–743. 18510489 10.1586/14779072.6.5.731

[61] Qiao Q, Gao W, Zhang L, Nyamdorj R, Tuomilehto J (2007) Metabolic syndrome and cardiovascular disease. Ann Clin Biochem 44: 232–263. 17456293 10.1258/000456307780480963

[62] Mottillo S, Filion KB, Genest J, Joseph L, Pilote L, et al (2010) The metabolic syndrome and cardiovascular risk. J Am Coll Cardiol 56: 1113–1132. 20863953 10.1016/j.jacc.2010.05.034

